# Hierarchical Effects of Lactic Fermentation and Grain Germination on the Microbial and Metabolomic Profile of Rye Doughs

**DOI:** 10.3390/foods12050998

**Published:** 2023-02-27

**Authors:** Walter Mancino, Paola Carnevali, Valeria Terzi, Pascual García Pérez, Leilei Zhang, Gianluca Giuberti, Lorenzo Morelli, Vania Patrone, Luigi Lucini

**Affiliations:** 1 Department for Sustainable Food Process (DiSTAS), Università Cattolica del Sacro Cuore, 29122 Piacenza, Italy; 2 R&D Food Microbiology & Molecular Biology Research Barilla G. e R. Fratelli S.p.A., 43122 Parma, Italy; 3 Council for Agricultural Research and Economics, Research Centre for Genomics and Bioinformatics, 29017 Fiorenzuola d’Arda, Italy; 4 Nutrition and Bromatology Group, Analytical and Food Chemistry Department, Faculty of Food Science and Technology, Universidade de Vigo, 32004 Ourense, Spain

**Keywords:** LAB, yeast, fermentation, rye, metagenomics, dough, metabolomics, germination

## Abstract

A multi-omics approach was adopted to investigate the impact of lactic acid fermentation and seed germination on the composition and physicochemical properties of rye doughs. Doughs were prepared with either native or germinated rye flour and fermented with *Saccharomyces cerevisiae,* combined or not with a sourdough starter including *Limosilactobacillus fermentum*, *Weissella confusa* and *Weissella cibaria.* LAB fermentation significantly increased total titrable acidity and dough rise regardless of the flour used. Targeted metagenomics revealed a strong impact of germination on the bacterial community profile of sprouted rye flour. Doughs made with germinated rye displayed higher levels of *Latilactobacillus curvatus*, while native rye doughs were associated with higher proportions of *Lactoplantibacillus plantarum.* The oligosaccharide profile of rye doughs indicated a lower carbohydrate content in native doughs as compared to the sprouted counterparts. Mixed fermentation promoted a consistent decrease in both monosaccharides and low-polymerization degree (PD)-oligosaccharides, but not in high-PD carbohydrates. Untargeted metabolomic analysis showed that native and germinated rye doughs differed in the relative abundance of phenolic compounds, terpenoids, and phospholipids. Sourdough fermentation promoted the accumulation of terpenoids, phenolic compounds and proteinogenic and non-proteinogenic amino acids. Present findings offer an integrated perspective on rye dough as a multi-constituent system and on cereal-sourced bioactive compounds potentially affecting the functional properties of derived food products.

## 1. Introduction

Rye bread is one of the most consumed cereal-based foods in northern Europe, China, and North America [1]. In these regions, rye (*Secale cereale* L.) is a valuable crop because of its resistance towards cold temperatures and northern climates [1].

From a nutritional perspective, rye flour is gaining attention for its health-promoting potential considering its hypocholesterolemic, anti-diabetic, anti-inflammatory, and cardioprotective properties [1]. Whole grain rye is characterized by a high content of dietary fibers, such as arabinoxylans and cellulose, and bioactive compounds with antioxidant properties, such as polyphenols [2]. Driven by consumer demand for sustainable and healthier products, the utilization of rye in cereal-based, functional foods has been widely explored [3]. In this context, seed germination or sprouting has gained popularity in cereal processing as an effective practice to improve grains’ nutritional quality and functional value. Sprouting involves the activation of endogenous hydrolytic enzymes that results in augmented digestibility of cereal proteins and starch [4]. Moreover, it increases the bioavailability of simple sugars, amino acids, phenolic compounds, minerals, and certain vitamins [4]. Likewise, sourdough fermentation is a traditional process that has been shown to affect different attributes of baked goods due to metabolic activities of indigenous yeasts and lactic acid bacteria (LAB). In addition, LAB can positively affect the nutritional value of fermented cereals by increasing the content of bioactive compounds, vitamins, and minerals, and decreasing the amount of anti-nutritional factors [5]. Lactic fermentation has emerged as a promising alternative to improve gut health, preventing digestion-related issues such as gluten sensitivity, and playing a role in the detoxification of common food mycotoxins [6,7,8].

Previous research had shown that both germination of grains before fermentation and the type of fermentation markedly affected the structure and the potential bioactive properties of rye constituents [9,10]. However, to the best of our knowledge, no comprehensive multi-omics study has yet been made to evaluate the impact of seed sprouting and lactic acid fermentation on rye dough composition and quality. Consequently, we took advantage of targeted metagenomics to assess the evolution of the inoculated starters and their overall impact on bacterial ecology. Untargeted metabolomics analyses were applied to uncover microbial contribution to the biochemical profile of grain doughs. We sought to unravel dynamic relationships between microorganisms and food matrix components and identify potential markers of functional capacity important for developing value-added rye products. The use of advanced metagenomics techniques can be important to verify and validate the ecological success of certain starters, which can be interesting for large-spectrum industrial productions [11]. In this context, this approach may be also of interest from an industrial perspective to gain further insight into the effect of traditional technologies such as germination and fermentation on rye flour microbial, chemical, and technological multiple changes aiming to encourage the production of newly rye-based wholesome ingredients and related food products. Finally, the study of oligosaccharide profiles as a function of fermentation, can give a fundamental contribution to understanding how the substrates are modified and which carbohydrate components are present within the dough system [12].

## 2. Materials and Methods

### 2.1. Microbial Strains and Growth Conditions

*Limosibacillus fermentum* UC3641, *Weissella confusa* UC4052, and *Weissella cibaria* UC405, previously isolated from sorghum sourdough [13], were used as LAB starters. The strains were grown in MRS medium in anaerobic conditions, using a jar and the anaerocult P reagent (Merck, Germany) at 37 °C. Cultures were harvested by centrifugation (4000× *g* × 10 min), washed twice with sterile saline solution (0.9% NaCl), and re-suspended in 5 mL of water. *Saccharomyces cerevisiae* was a commercial, compressed fresh baker’s yeast (Lessafre, Marcq-en-Barœul, France), reintegrated in water before use.

### 2.2. Germination of Rye Flour and Micro-Malting Process

Unprocessed commercial rye grains (SU Bendix winter rye) were subjected to a preliminary sieving step. Kernel size fractions between 2 and 2.5 mm were obtained using an Octagon 200 test sieve shaker (Endecotts Ltd., London, UK). Malting was performed on 100 g sieved seed batches with an Automatic Micromalting System (Phoenix Biosystems, Adelaide, SA, Australia) (Figure S1). The following malting cycle (144 h in total) was applied: 15-min wash at room temperature; 7-h and 15-min steep at 15 °C; 8-h rest at 19 °C; 9-h steep at 15 °C; 6-h germination at 19 °C; 30-min steep at 15 °C; 88-h and 30-min germination at 15 °C; 7-h kilning from 30 to 40 °C; 6-h kilning from 40 to 60 °C; 6-h and 30-min kilning from 60 to 70 °C; 4-h and 30-min kilning from 70 to 80 °C; and 30-min kilning at 25 °C. The chemical composition of native rye flour was: dry matter (DM): 90.2 g/100 g; total starch: 59.0 g/100 g DM; crude protein: 9.3 g/100 g DM; total dietary fiber: 17.6 g/100 g DM, free glucose: 0.28 g/100 g DM. The chemical composition of sprouted rye flour was: DM: 93.1 g/100 g; total starch: 49.3 g/100 g DM; crude protein: 11.1 g/100 g DM; total dietary fiber: 24.1 g/100 g DM, free glucose: 5.6 g/100 g DM.

### 2.3. Dough Preparation and Chemical-Physical Characterization

In brief, 250 g of native or sprouted rye flours were mixed with 250 mL of tap water and 1% (*w*/*v*) of NaCl. The kneading process was preformed through a commercial kneading machine (IMETEC ZERO-GLU KM 1500, Tenacta Group Spa, Azzano S. Paolo, Italy), initially without inoculum, at machine speed 1 for 2 min. As for fermentation, three different experimental conditions were tested: (i) *S. cerevisiae* fermentation, in which *S. cerevisiae* was inoculated at a final concentration of 2% (*w*/*w*), SC; (ii) mixed fermentation, in which a mix of the three LAB strains (10^9^ CFU/mL) plus *S. cerevisiae* (2% *w*/*w*) was added to the dough, LAB + SC; and (iii) spontaneous fermentation, where non-inoculated doughs were prepared by replacing the inoculum with an equal volume of plain water, considered as the control. The initial concentration of LAB in each inoculated dough was between 1.89–3.00 × 10^7^ cfu/g, as expected, whereas the initial concentration of *S. cerevisiae* in inoculated doughs was 1.30–1.68x10^6^ cfu/g. After inoculation, the kneading process was carried out for 5 min at machine speed 3. All doughs were maturated for 24 h; for the mixed fermentation, an initial maturation with only inoculated LAB was performed for 8 h (30 °C and 60% relative humidity), after which yeast was added and the dough was left to leaven for the remaining 16 h (35 °C and 65% relative humidity). The same temperature and humidity conditions were applied for SC and control. Dough samples were prepared in duplicate and fermentations were repeated twice. All the doughs were analyzed for pH (pH meter Hanna Edge), total titratable acidity (TTA) [14], water activity (a_w_) (Aqualab Serie 4; Steroglass, Perugia, Italy), dough rise, total yeast, and LAB count. Dough rise was determined as follows: after mixing, 20 g of the dough was transferred into a graduated cylinder. The height of the dough was measured before and after fermentation, and the dough increase in volume was calculated as previously reported [15]. Yeast and LAB counts were performed in duplicate on YPD agar supplemented with chloramphenicol and MRS agar with 1% of cycloheximide, respectively.

### 2.4. Extraction of DNA and Full-Length 16S Metagenomics Analysis

Microbial cells were harvested from doughs as previously described [16]. Total DNA was extracted from bacterial pellets using the Fast DNA Spin Kit for Soil (MP biomedicals, Irvin, CA, USA) according to the protocol supplied. DNA quantity was determined by a Qubit fluorimeter (Life Technologies, Carlsbad, CA, USA), while DNA integrity was checked through 0.8% agarose gel electrophoresis. Samples were prepared according to the guidelines for preparing SMRTbell template for sequencing on the PacBio Sequel I System (PacBio, Menlo Park, CA, USA) at Macrogen (Seoul, Republic of Korea). The library was constructed with SMRTbell^®^ Express Template Prep Kit 2.0 including 27F-1492R primers [17] along with barcode according to the manufacturer’s instructions (Pacific Biosciences, Menlo Park, CA, USA); library purification was carried out using Ampure^®^ PB bead (Pacific Biosciences, Menlo Park, CA, USA). Purified SMRTbell library from the pooled and barcoded samples was sequenced on a single PacBio Sequel cell using a SMRT Cell 1M v3 Tray. The resulting data were processed using the rDNATools pipeline [18]. Ambiguous reads and short reads (<1199 bp) were filtered out, and extra-long tails were trimmed according to the target size of the bacterial 16S rRNA gene. Chimeras were identified and removed using the software Vsearch v2.14.2 using the default settings. Then, a distance matrix was generated using the Unweighted/Weighted UniFrac distance, and reads were clustered using the average neighbor method. Operational Taxonomy Unit (OTU) picking was based on the de novo method; sequences that shared over 99% similarity were assigned to a single OTU. Taxonomic assignment of OTUs was obtained using QIIME2 bioinformatic pipeline v 2022.2 [19], which provides functionality for working with and visualizing taxonomic annotations of features. OTUs were aligned with the representative sequence of the NCBI 16S Database with a similarity cutoff of 97% for species differentiation.

### 2.5. Oligosaccharide Semi-Quantification of Rye Doughs

The oligosaccharide semi-quantification of the different rye doughs was performed by high-performance anion exchange chromatography coupled with pulsed amperometric detection (HPAEC-PAD) approach. All samples were subjected to a previous extraction, in which 1 g of sample was mixed with 10 mL of deionized water, and the resulting mixture was homogenized by a high-speed rotor (Polytron PT 1600-E) for 1 min and centrifuged at 8000× *g* for 10 min at 4 °C (Eppendorf 5810R, Hamburg, Germany). The supernatants were collected, syringe filtered (0.22 µm pore size), and transferred into vials for the subsequent analysis. The experimental conditions applied for HPAEC-PAD analysis were previously described [20]. The equipment employed consisted of a Dionex ICS-5000+ (Thermo Fisher Scientific, Waltham, MA, USA), containing an electrochemical cell with a gold working electrode combined with a pH-Ag/AgCl reference electrode as detection system. The chromatographic separation was achieved through a Dionex CarboPac PA200 column (3 × 250 mm) coupled to a guard column (3 × 50 mm), as the stationary phase (both purchased from Thermo Scientific), which provides a high-resolution separation of monosaccharides and linear oligosaccharides. The mobile phase consisted in a binary solvent system that included 100 mM NaOH (eluent A) and 1 M sodium acetate in 100 mM NaOH (eluent B). The experimental runs presented a total time of 75 min, the flow rate was adjusted at 0.4 mL min^−1^ and temperature for both column and detector compartments were set at 27 °C, following a multi-gradient elution system: 0–10 min, 98% A; 10–35 min, 55% A; 65–75 min, 98% A. The amperometric detector was set in terms of several potentials and durations as follows: E1 = 0.10 V (t1 = 0.40 s); E2 = −2.00 V (t2 = 0.01 s); E3 = 0.60 V (t3 = 0.01 s); E4 = −0.10 V (t4 = 0.06 s). The semi-quantification of oligosaccharides was achieved according to their polymerization degree (PD), with respect to individualized standards that are representative of three different well-separated structural classes: monosaccharides, low-PD oligosaccharides, and high-PD oligosaccharides. Thus, xylose was applied as the reference standard for monosaccharides (y = 3.8348x + 1.007, R^2^ = 0.9918), arabinotriose was selected as the reference standard for low-PD oligosaccharides (y = 3.7960x + 1.656, R^2^ = 0.9878), and arabinooctaose was chosen as the reference standard for high-PD oligosaccharides (y = 0.9963x + 0.8871, R^2^ = 0.9842). The results were expressed as carbohydrate content in mg g^−1^ of sourdough. The standards and reagents employed for oligosaccharide profiling were purchased from Sigma Aldrich^®^, Darmstadt, Germany (xylose) and Megazyme^®^, Bray, Ireland (arabinotriose and arabinooctaose).

### 2.6. Untargeted Metabolomic Profiling of Rye Doughs

The untargeted metabolomic profiling of the different rye doughs was obtained through an ultra-high performance liquid chromatography instrument (Agilent 1200 series), presenting a binary pump and JetStream electrospray source, coupled to a quadrupole time-of-flight mass spectrometer (UHPLC/QTOF-MS; Agilent iFunnel 6550). Before the analytical determination, 1 g of each rye doughs was mixed with 10 mL of the extraction solvent MeOH:H_2_O:HCOOH (80:19.9:0.1, *v*/*v*/*v*) and the extraction was performed under the same conditions described earlier [21]. All analytical conditions were set as described elsewhere [21,22]. Briefly, the injection volume was 6 µL, the chromatographic separation was achieved through an Agilent Zorbax Eclipse Plus C18 column (50 × 2.1 mm, 1.8 µm), applying an AcN:H_2_O binary mobile phase with a gradient elution: 6%–94% organic phase for a 33 min run, with a flow rate of 200 µL min^−1^. The analytical conditions for the QTOF performance were employed as follow: N_2_ as sheath gas with a flow of 10 L min^−1^ at 350 °C, drying gas was applied with a flow of 8 L min^−1^ at 330 °C, nebulizer pressure was set at 60 psi, nozzle voltage at 300 V, and capillary voltage at 3.5 kV. The mass spectrometer was adjusted in positive polarity and SCAN mode, with a detection range of 100–1200 *m/z*, with a nominal resolution of 40,000 FWHM. Moreover, quality control pooled samples were obtained and further analyzed through data-dependent MS/MS mode (12 precursors per cycle at 1 Hz, 50–1000 *m/z*, positive polarity), applying different collision energies: 10, 20 and 40 eV. The acquired raw data were later processed by the Agilent MassHunter Profinder v.10.0 using the “find-by-formula” algorithm, through mass and retention time alignments (5-ppm and 0.05 min tolerance, respectively). The annotation of the obtained chemical features was based on their accurate mass and isotopic patterns, given by the exact masses, relative abundance and *m/z* spacing, using the FooDB database (available at foodb.ca) to achieve their identification. Data reduction was achieved by applying the “filter-by-frequency” feature, exclusively retaining the features observed in all the replicates within the same treatment. As a result, the untargeted compound annotation obtained was in compliance with identification level 2 provided by the COSMOS Metabolomics Standard Initiative (putatively annotated compounds). To improve the confidence in the compound annotation raw data were processed by MS-DIAL software (v. 4.90), achieving the identification of chemical features through MS-MS spectral data, according to mass accuracy data, mass isotopic patterns, and spectral alignment matching. The parameters set for identification were: retention time range, 1–32 min; mass range, *m/z* 80–1200; mass tolerance, 0.05 Da. For data reduction, a filter step was performed, removing the identities that were not acquired in, at least the 80% of replicates. Finally, a score cut-off of 80% was selected to retain those compounds with the highest identification fidelity according to MS^2^ level. All chemicals used for extraction and chromatographic equipment were LC-MS grade, purchased from VWR (Milan, Italy).

### 2.7. Statistics

A two-way analysis of variance (ANOVA) was performed to analyze the effect of starter and germination on dough general parameters using GraphPad Prism version 8.0.0 for Windows (GraphPad Software, San Diego, CA, USA). Metagenomic data were processed using the QIIME2 v.2020.2 platform for diversity analysis of dough microbial communities. Observed species, Chao1, Shannon and Gini-Simpson indices were calculated to assess within sample diversity; sequencing depth was characterized by Good’s coverage. Weighted and unweighted Unifrac distances were calculated, and Principal Coordinate Analysis (PCoA) was performed on the distance matrices to visualize community variation across samples. The OTU table was uploaded to the Microbiomeanalyst server for compositional profiling and comparative analysis, using 10% prevalence in samples for the low count filter, the default settings for other filtering and total sum scaling for data normalization [23]. To test for significance in differential bacterial taxa abundance according to starter and flour, respectively, the algorithm DeSeq2 was used [24]. The name of the samples was as follows: nDLY (native rye–Dough–LAB + SC); sDLY (sprouted rye–Dough–LAB + SC); nDY (native rye–Dough–SC); sDY (sprouted rye–Dough SC); nDCTR (native rye–Dough–Control); sDCTR (sprouted rye–Dough–Control); nF (native rye–Flour); sF (sprouted rye–Flour). For both oligosaccharide and untargeted metabolomics profiling approaches, all sourdough samples were extracted in triplicate, and two technical determinations were carried out for each replicate (*n* = 6). The results for carbohydrate content of rye sourdoughs were statistically analyzed by one-way ANOVA followed by Duncan’s post hoc test, setting a significance value of α = 0.05, using the software SPSS 25 (IBM). The Agilent Mass Profile Professional v. 15.1 software analyzed the metabolomics data as previously indicated compounds were filtered by abundance, log2-transformed, and normalized at the 75th percentile [22]. The abundance of each compound was further baselined against the median abundance among all samples. Afterwards, an unsupervised multivariate hierarchical cluster analysis (HCA) was performed to evaluate the similarities and dissimilarities of all factors as a function of their metabolic profile (Euclidean distance, Ward’s linkage rule). Later, a Chemical Similarity Enrichment Analysis for Metabolomics (ChemRICH, available at chemrich.fiehnlab.ucdavis.edu) was performed to define the chemical composition of sourdoughs due to the addition of LAB. To that aim, compounds were filtered on Volcano plot and only the compounds showing a significantly different accumulation between treatments (*p* < 0.05) and with fold-change values > 2 were considered [25]. Finally, a supervised multivariate orthogonal projection to latent structures discriminant analysis (OPLS-DA) was carried out by the SIMCA v. 16.0.2 software (Umetrics). Model quality was evaluated according to goodness-of-fit parameters (R^2^X and R^2^Y), and goodness-of-prediction parameter (Q^2^Y). OPLS-DA predictive models were further statistically validated by cross-validation ANOVA (CV-ANOVA), and model overfitting was discarded through the development of permutation tests (*n* = 100). Such approach was followed by a variable importance in projection (VIP) analysis, providing insight into the compounds exhibiting the highest influence on the discrimination between treatments, known as VIP markers, according to their given VIP score [22].

## 3. Results

### 3.1. General Parameters of Rye Doughs

Key technological parameters of the different doughs after fermentation are presented in Table 1. Rye doughs fermented with LAB + yeast showed total LAB counts reaching approximately 10 log CFU/g after 24-h fermentation; this value significantly exceeded the numbers of total LAB in either SC or control rye doughs, suggesting the actual growth of inoculated LAB starters. No significant difference in final LAB abundance was found between native and sprouted rye flour. A similar trend was observed for total yeast counts. Before fermentation, the pH was 6.23 ± 0.04 for doughs made with native flour and 5.88 ± 0.07 for doughs with sprouted rye flour, respectively.

As expected, the addition of LAB starters caused a decrease in the pH value to about 4 both in sprouted and native rye doughs, as compared to either SC or control. However, the difference between final pH values was significant only when comparing LAB + SC vs. SC (*p* = 0.002) in sprouted rye samples. Consistently, the application of LAB starters significantly increased total acid concentration in rye doughs as compared to either yeast or control (Table 1). A two-way ANOVA revealed that there was a significant interaction between the effects of starter and germination as concerns TTA value (*p*-value interaction < 0.0001). Notably, the TTA level of LAB + SC doughs made with sprouted rye flour was higher than that of the corresponding doughs produced from native rye flour (2.08 ± 0.05 vs. 0.82 ± 0.06, *p*-value < 0.0001). The inclusion of LAB as a starter for rye flour fermentation displayed no significant impact on dough a_w_. Significant differences were observed for yeast (alone or in combination with LAB) versus control in native rye flour doughs (*p* = 0.0004 and *p*-value = 0.006, respectively). Concerning dough volume, LAB seemed to contribute to dough rise during leavening markedly. The volume increase was higher for LAB + SC vs. SC or control regardless of the type of rye flour used; differences were all statistically significant but for LAB + SC vs. SC in native rye dough (220% ± 18.87% vs. 143.3% ± 4.71%; *p*-value = 0.01). Analogously to a_w_, statistical analysis indicated that the type of fermentation had the same effect in sprouted and native rye doughs (*p*-value = 0.22).

### 3.2. Diversity of Bacterial Communities in Rye Doughs

PacBio SMRT sequencing of the complete bacterial 16S rRNA gene resulted in 167,207 total filtered high-quality reads, with numbers ranging from 4,188 to 16,844 reads per sample (mean 10,450 reads). Clustering to 99% similarity yielded 5,657 distinct OTUs; the mean number of OTUs per sample was 353 (range 58–609; Table 2). To assess sample diversity, different indexes were calculated including Chao1, Shannon, and Gini-Simpson (Table 2).

**Figure 1:**
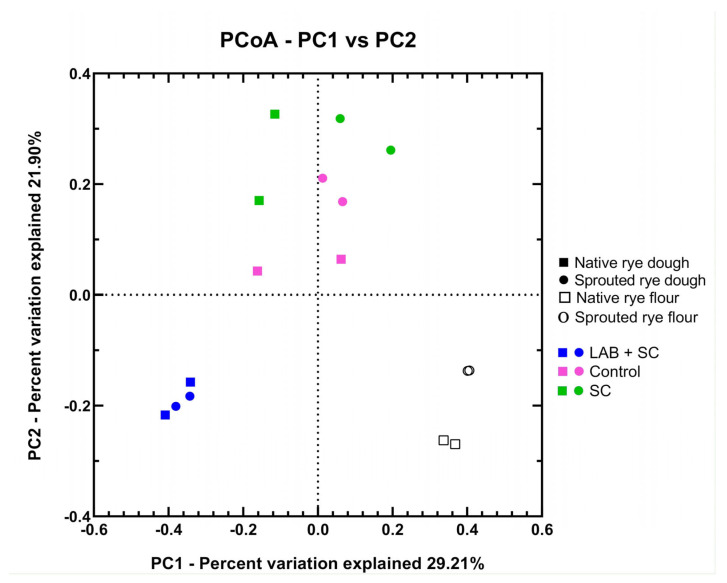
Beta-diversity analysis of rye dough and flour samples. PCoA plot based on unweighted UniFrac distances of microbial communities among all samples. Solid symbols represent doughs made with native rye flour (squares) and sprouted rye flour (circles); empty symbols represent native rye flour (squares) and sprouted rye flour (circles). Dough samples were colored according to the starter used for fermentation: blue = LAB + SC, green = SC, pink = no inoculated starter.

No significant differences were found among dough samples in any alpha diversity indexes. As expected, flours displayed the highest bacterial diversity among all tested samples, suggesting that fermentation exerted a selection pressure on the community structure of the dough microbiota (Table 2). Overall, native rye flour and doughs tended to have a lower bacterial richness as compared to their counterparts obtained from sprouted rye. Good’s coverage ranged from 97% to 99% suggesting that a high percentage of the total species was represented in each sample (Table 2). Beta diversity, based on an unweighted UniFrac distance matrix, highlighted three distinct bacterial clusters in the PCoA plot (Figure 1). Differences in microbial composition between the samples allowed a clear discrimination between flour samples and dough samples obtained by mixed fermentation, respectively. A further group, including both spontaneously fermented doughs and doughs produced by *S. cerevisiae* fermentation, could be identified.

### 3.3. Bacterial Taxonomic Composition of Dough Samples

**Figure 2:**
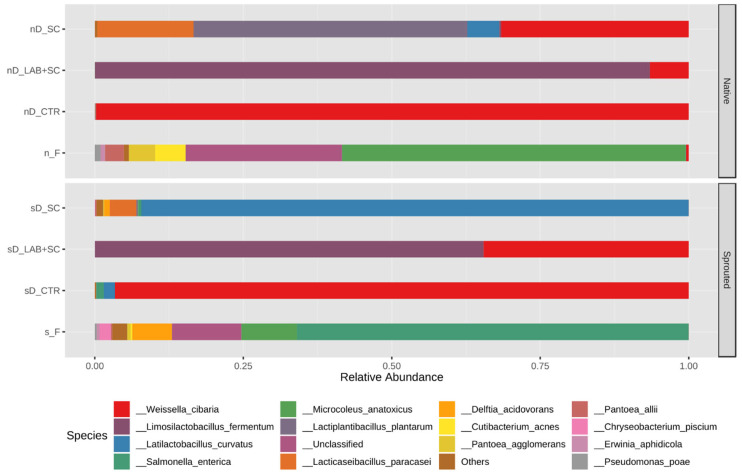
Relative abundance of bacterial species found in dough and flour samples. The bar plot represents the top 15 most abundant taxa among all samples identified to the species level. “Others” refers to merged species that individually showed a relative abundance below 0.25%. “Unclassified” are bacterial taxa not identified at the phylum level. The results are expressed as average of replicates for each type of dough. In the *x*-axis are reported the name of the samples as follow: sD, sprouted rye dough; nD, native rye dough; s_F, sprouted rye flour; n_F, native rye flour, CTR, dough control; LAB + SC, dough fermented with LAB + SC; SC, dough fermented with SC.

More than 93% of the sequences obtained by the PacBio SMRT sequencing were associated with known taxa; unclassified reads were 6.5% of the total sequences. *Weissella*, *Limosillactobacillus*, *Salmonella*, *Latilactobacillus,* and *Microcoleus* were the dominant genera in the metagenomic dataset, with an average abundance of 28.48%, 19.89%, 13.86%, 12.53%, and 6.36% of the total reads, respectively. At species level, reads were assigned to 56 different taxa, and the species with an average greater than 0.25% were 12 taxa (Figure 2). The bacterial community composition of flours differed between sprouted and native rye. *Salmonella enterica* represented more than 65% of bacterial microbiota in sprouted rye flour followed by *Microcoleus anatoxicus*, *Delftia acidovorans*, and others (Figure 2). Conversely, the most abundant bacterial species in native rye flour was the cyanobacterium *Microcoleus anatoxicus*, accounting for about 43% of the total sequences; less common species included *Pantoea agglomerans* and *Cutibacterium acnes*.

Our results indicated that, sprouted rye doughs had greater uniformity in the bacterial community structure for all the 3 fermentation conditions than the native rye counterparts. *L. fermentum* and *W. confusa/cibaria* were the only bacterial species detected in dough samples inoculated with the lactic acid starter. As expected, the two closely related *Weissella* species were indistinguishable using 16S rRNA gene sequencing. *L. fermentum* sequences greatly outnumbered those ones classified under the species pair *W. confusa*/*W. cibaria* (Figure 2), reaching 93.56 ± 4.73% in native rye doughs, and 65.26 ± 0.27% in sprouted rye doughs. Yeast-leavened doughs were enriched in cereal-sourced LAB with a different species composition based on the rye flour used. *Latilactobacillus curvatus* was dominant (relative abundance > 89%) in sprouted rye doughs, while *Lacticaseibacillus paracasei* and *Latilactobacillus graminis* were found at low relative abundance. Native rye doughs harbored a bacterial community consisting of *Lactoplantibacillus plantarum* (with an average of 42.6%) followed by *Lacticaseibacillus paracasei* (relative abundance > 16%)*; Lactobacillus curvatus* was detected with a relative abundance over 4%, while *W. confusa/cibaria* accounted for 38.5% of total sequences in nDY (Figure 2). Control doughs resulted in a microbiota that differed from that of other doughs and was broadly dominated by indigenous *W. confusa/cibaria* strains.

### 3.4. Differential Analysis of Discriminant Species between Dough Samples

**Figure 3:**
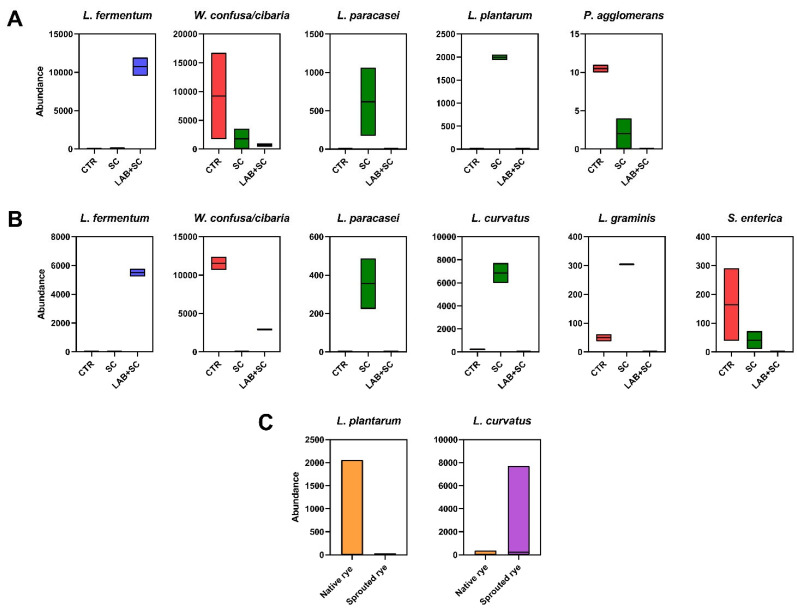
Differential abundance analysis of bacterial species among rye doughs. Box and whiskers plot indicate the proportion of differentially abundant taxa between starters used for fermentation in native rye doughs (**A**) and in sprouted rye doughs (**B**), and between native and sprouted rye doughs (**C**). Only significant species (FDR < 0.05) detected by DESeq2 are reported in the figure. Boxes represent the minimum and maximum of abundance values of replicates for each condition; the line in the box is the median.

Differential analysis of the abundance of microbial species revealed several features that varied significantly according to either starter or germination. When considering the impact of starter, *L. fermentum* was higher in LAB + SC samples compared to either SC or control samples, regardless of rye germination (Figure 3A,B). As for native rye doughs (Figure 3A), control samples were enriched in *W. confusa/cibaria* and *P. agglomerans* with respect to native doughs inoculated with LAB and yeast.

Moreover, the species *L. plantarum* and *L. paracasei* were more abundant in yeast-leavened samples than doughs obtained by mixed fermentation, or native spontaneous fermentation samples. Among sprouted rye doughs, spontaneously fermented samples displayed significantly higher levels of *W. confusa/cibaria* and *S. enterica* than those observed in SC samples and in LAB + SC samples, respectively. Notably, the inoculation *S. cerevisiae* alone in sprouted rye samples was associated with higher proportions of endogenous LAB species including *L. curvatus*, *L. graminis* and *L. paracasei*. In fact, these species resulted in being higher in abundance in SC samples with respect to both LAB + SC samples and control samples. As regards the comparison between sprouted and native rye flours, *L. curvatus* was significantly higher in sprouted rye doughs as compared to native rye doughs (*p*-value = 0.045). Conversely, *L. plantarum* was significantly higher in native rye samples to sprouted samples (*p*-value = 0.045).

### 3.5. Oligosaccharide Semi-Quantification of Rye Sourdoughs

**Figure 4:**
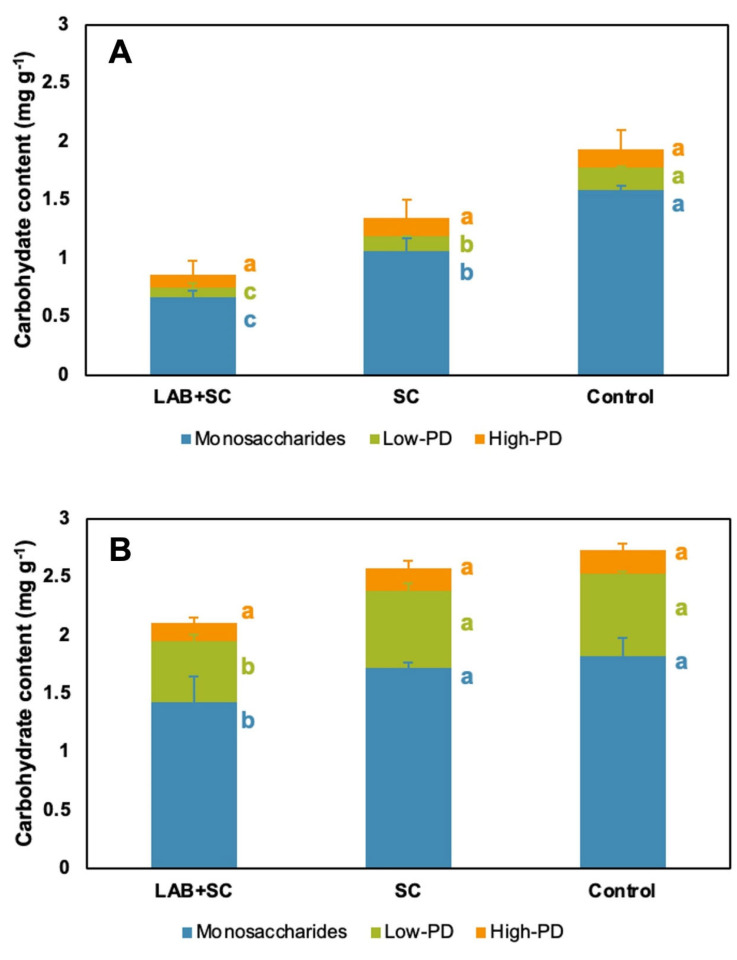
HPAEC-PAD carbohydrate content of rye sourdoughs. Carbohydrate content of native rye doughs (**A**). Carbohydrate content of sprouted rye doughs (**B**). Results are expressed as carbohydrate content (mg g-1) referred as xylose, arabinotriose, and arabinoctaose equivalents for monosaccharides, low-polymerization degree oligosaccharides (low-PD), and high-polymerization degree oligosaccharides (high-PD) contents, respectively (**B**). Vertical bars indicate standard deviation (*n* = 6). Different letters indicate statistically significant differences (*p* < 0.05) among treatments.

The oligosaccharide semi-quantification of rye flour doughs is shown in Figure 4. In general, germination played a critical role on carbohydrate compositions since native rye doughs exhibited a lower carbohydrate content to the sprouted counterparts. In parallel, adding fermentation starters played a significant role on the carbohydrate content of rye doughs (Figure 4).

Thus, for native rye doughs, SC-mediated fermentation promoted a significant decrease of both monosaccharides and low-PD oligosaccharides that were further significantly decreased in the LAB + SC fermentation by whereas high-PD carbohydrates were not affected (Figure 4A). In total, the combination of LAB with SC led to a harsh carbohydrate content reduction of 55.5% with respect to control. In contrast, the fermentation of sprouted rye doughs caused only a significant decrease in the carbohydrate content in the case of mixed fermentation, as SC fermentation did not promote any significant difference in comparison to control (Figure 4B). As a result, concerning total carbohydrate content, the LAB + SC treatment led to a 22.8% decrease with respect to control, suggesting a lower impact of fermentation than that observed for native-derived dough. Concerning the different carbohydrates, the high-PD oligosaccharides content was not affected by the type of fermentation, whereas the contents of both monosaccharides and low-PD oligosaccharides was significantly decreased after the LAB + SC combined fermentation (Figure 4B).

### 3.6. Untargeted UHPLC/QTOF-MS Metabolomic Profile of Rye Doughs

**Figure 5:**
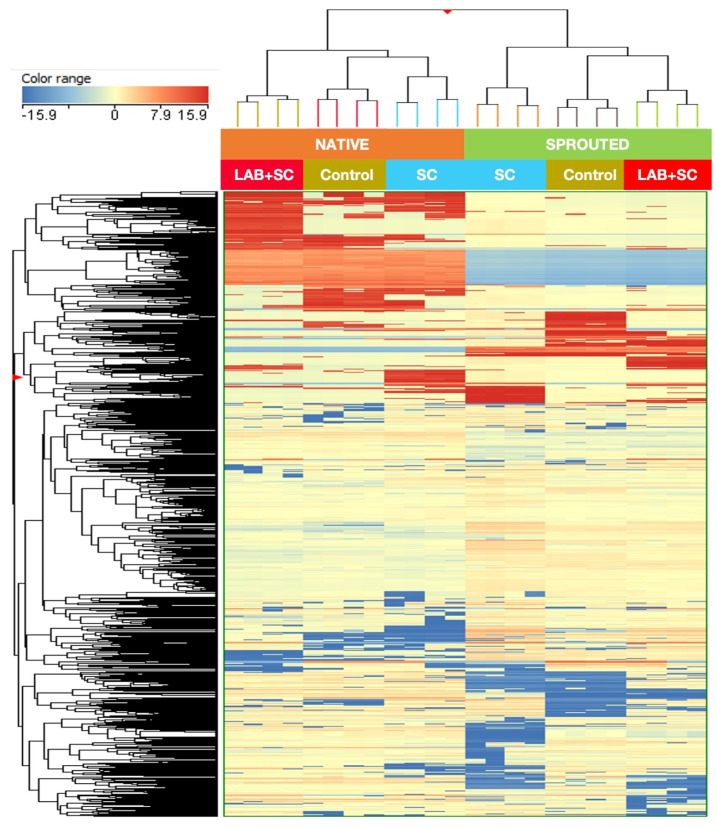
Unsupervised hierarchical cluster analysis on the untargeted metabolic profiling of rye dough. The fold change values, represented by a color range, for each compound were calculated with respect to the median of all samples, and further used to obtain a fold change-based heatmap, according to Ward’s algorithm (Euclidean distance). The factors involved in clustering were NATIVE and SPROUTED, for native and sprouted-derived rye sourdough, respectively; and control, SC, and LAB + SC, for unfermented, yeast-fermented and yeast and LAB-combined fermented rye sourdough, respectively.

Rye doughs were subjected to metabolic profiling via UHPLC/QTOF-MS, providing 1909 annotated chemical features (Table S1). From these annotated compounds, 158 were identified according to their MS^2^ spectral features (Table S2). The effect of grain germination and the addition of starters on the metabolic profile of rye doughs were evaluated by an unsupervised multivariate hierarchical cluster analysis (HCA), and the results are displayed in Figure 5. Among the factors involved in this study, germination was the most prevalent factor affecting the metabolome of samples since it ruled the establishment of two major clusters. Secondarily, within both clusters, fermentation starters played a significant role, providing three subclusters according to the different experimental conditions involved in dough production: non-inoculated, SC, and LAB + SC (Figure 5).

**Figure 6:**
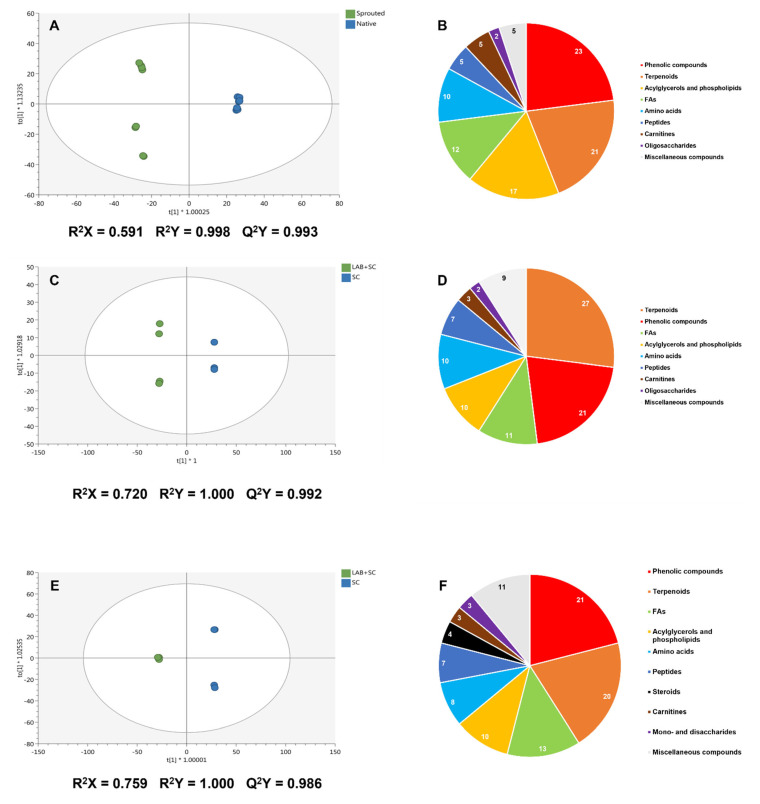
Orthogonal projection to latent structures discriminant analysis and variable importance in projection analysis of rye doughs. The OPLS models were combined with the proportion of VIP markers on the discrimination of metabolomic effects caused by germination conditions (**A**,**B**), fermentation starters on native rye doughs (**C**,**D**), fermentation starters on sprouted rye doughs (**E**,**F**).

Due to the heterogeneous metabolic profile of fermented rye doughs, an additional supervised multivariate orthogonal projection to latent structures discriminant analysis (OPLS-DA) was performed. It was followed by a variable importance in projection (VIP) analysis, with the aim of discriminating the effect of germination and starters on the metabolome of these matrices, providing insight on the metabolic markers mostly involved in such discrimination (VIP markers). The Figure 6 shows the OPLS-DA models and the proportion of VIP markers according to their chemical class for the discrimination between germination conditions (Figure 6A,B, respectively), fermentation starters on native rye-derived doughs (Figure 6C,D, respectively), and fermentative starters on sprouted rye-derived doughs (Figure 6E,F, respectively).

Moreover, the full list of VIP markers associated with all models, together with their VIP score, logFC values, and chemical class are provided in Tables S4–S6, respectively. In all cases, the obtained OPLS-DA models showed high-quality parameters in terms of goodness-of-fit, given by R^2^X and R^2^Y coefficients, and predictability, given by the Q^2^Y coefficient (Q^2^Y > 0.5; Figure 6). Concerning germination, the OPLS-DA model spotted a definitive role of this factor on the metabolome of rye doughs, indicating a clear discrimination between native rye- and sprouted rye-derived doughs (Figure 6A). Phenolic compounds, terpenoids, and phospholipids (Figure 6B) predominantly represent the VIP markers provided for the discrimination between native and sprouted-derived doughs. In general, and regarding the logFC values (Table S3), sprouted rye-derived doughs exhibited an enhanced accumulation of phenolic compounds, including flavonoids like mulberrin, zapotinin, and isoferreirin (logFC = 13.5), and phenolic acids, mostly represented by spermidine esters, and resorcinols. In the case of terpenoids, triterpenoid acids like 3-benzoyloxy-6-oxo-12-ursen-28-oic acid and 2, 3, 23-triacetylsericic acid (log FC = 13.5) were accumulated in sprouted rye-derived doughs (Table S3). To a lesser extent, amino acids like His and Phe derivatives and oligopeptides, as well as glucosinolates were found to be differentially up accumulated in sprouted rye doughs, providing evidence on the metabolic richness of this matrix. Conversely, the accumulation of lipid metabolites did not follow a clear pattern. Metabolites like docosan-1-ol, phosphatidylethanolamines, and 3-hydroxy-9-hexadecenoylcarnitine exhibited a decrease in native rye doughs (log FC = ™9.4; Table S3), whereas saturated fatty acids 22-hydroxydocosanoate and 10-hydroxymyristic acid methyl ester and some di-glycerides were mostly measured in sprouted rye doughs (log FC = 13.5; Table S3). As germination played such a discriminant effect on the metabolome of rye sourdoughs, two additional OPLS models were performed to evaluate the impact of starters on either native rye or sprouted rye doughs (Figure 6C,E, respectively). In both cases, a clear discrimination between SC-fermented doughs and LAB + SC-fermented doughs was obtained, and phenolic compounds, terpenoids, and lipid metabolites were mostly identified as VIP markers (Figure 6D,F, respectively). Firstly, in the case of native rye doughs, the inoculation of LAB promoted a general up-regulation of the metabolome, since 54% of VIP markers possessed logFC = 8.6, and only 17% of markers were found to be down-accumulated as compared to SC-fermented doughs (Table S4). Thus, terpenoids were generally accumulated, ranging from triterpenoids and sesquiterpenoids to monoterpenoids, including sterols and carotenoids (Table S4). Likewise, phenolic compounds were all up-accumulated due to LAB addition, involving flavonoids, phenolic acids like *p*-coumaroyl derivatives, and spermine and putrescine esters, stilbenes, coumarins and lignans (Table S4). In parallel, amino acids were also selected as VIP markers accumulated in LAB + SC fermented doughs, represented by both proteinogenic amino acids, such as Gln (logFC = 3.3), Gly (logFC = 3.4), and Cys derivatives (log FC = 8.6), and non-proteinogenic amino acids, like ornithine (logFC = 2.0; Table S4). Peptides were found to be accumulated as a result of LAB inclusion (log FC = 2.3–8.6; Table S4) as well, suggesting an intense proteolytic activity. In contrast, lipid metabolites showed an unclear pattern of accumulation between LAB + SC fermentation and SC fermentation, as given by log FC values. Notably, lysophospholipids were mainly accumulated in LAB + SC-fermented doughs (log FC = 2.0–8.6), whereas fatty acids were heterogeneously detected (Table S4). A similar trend was observed for the inclusion of LAB as fermentation starters on the metabolome of sprouted rye doughs, as indicated by the corresponding OPLS-DA (Figure 6E). A metabolic stimulation was shown by the inclusion of LAB, with 76% of VIP markers up regulated in LAB + SC-fermented matrices to those fermented exclusively with SC (Table S5). Again, phenolic compounds constituted the class with the highest contribution to VIP markers, followed by terpenoids and lipid metabolites (Figure 6F). Considering phenolic compounds, lignans were the compounds presenting the highest accumulation (log FC = 10.0 for schidigeragenin B and clusin, Table S5), together with ferulic, caffeic acid esters (log FC = 10.0), whereas flavonoids presented much lower log FC values (Table S5). Considering terpenoids, LAB inclusion promoted the accumulation of high-isoprene subunits terpenoids, including steroids, triterpenoids and sesquiterpenoids (log FC = 10.0, Table S5), whereas the accumulation of mono- and diterpenoids was reduced (log FC < −3.3). In parallel, in the case of amino acids and peptides, the accumulation and down-accumulation did not follow a clear pattern. While saturated fatty acids, especially octadecanoic acids (log FC = −12.8), and sulfur-containing compounds, like 1-methoxyspirobrassinin (log FC = −12.8), were harshly down accumulated upon the addition of sourdough LAB starters, lysophospholipids were found accumulated upon the inclusion of LAB (log FC = 3.7–10.0; Table S5).

## 4. Discussion

This study explored the microbial, chemical, and technological profiles of rye doughs made with either native or sprouted flour and fermented with *S.cerevisiae* in combination or not with selected LAB starters. Two complementary approaches were applied to assess the metabolic profiling of rye doughs after fermentation. Firstly, the carbohydrate profile of rye doughs was assessed, and results indicate that grain germination and LAB fermentation played a significant role in the composition of these constituents. The carbohydrate content of sprouted rye doughs was significantly higher than that of native rye doughs, due to the induction of hydrolytic enzymes during seed germination, which includes amylases, pentosanases and glucanases [26]. Due to hydrolytic activity, insoluble fiber is mainly converted into soluble sugars, such as monosaccharides, that were spotted as the major sugar constituents of rye doughs in this work. Notably, VIP analysis indicated that characteristic oligosaccharides of sprouted rye doughs were maltopentaose and maltotetraose, functional maltodextrins potentially involved in glycemic control response and enterocyte differentiation [27]. Considering the fermentation starters, including LAB promoted a significant decrease in carbohydrate content in terms of monosaccharides and low-PD oligosaccharides. This can be explained considering the heterofermentative metabolism of sourdough LAB, which relies on the activity of a wide range of catabolic enzymes [7]. Analysis of technological parameters revealed important features of experimental doughs connected to the evolution of bacterial ecology during fermentation and the interplay between starter inoculation and germination. As expected, the application of sourdough LAB starters led to a substantial reduction of pH, especially in comparison to yeast-leavened doughs, as a result of LAB extensive exploitation of carbohydrates for organic acids biosynthesis [28,29]. Indeed, the germination-related enzymatic breakdown of carbohydrates into simple sugars can boost fermentative metabolism by sourdough LAB resulting in the accumulation of organic acids [9,30]. Interestingly, LAB + SC fermentation was also associated to a greater dough rise as compared to either SC or control. Heterofermentative LAB activity can affect dough leavening through the production of CO_2_ [31]. In mixed LAB + SC samples, metagenomics analysis highlighted a strong dominance of inoculated *L. fermentum* over *Weissella* strains at the end of the fermentation. On the other hand, endogenous *W. confusa/cibaria* was the predominant taxon in control samples. The latter finding is not surprising since several studies identified *Weissella* alone, or in combination with other LAB, as the dominant bacterial genus in rye sourdough after 24 h fermentation which may include or not refreshments [31,32,33,34,35]. Indeed, the ecological fitness of sourdough microorganisms is largely dependent on the interplay between strain-specific traits and process conditions including temperature, pH, dough hydration, fermentation time, and type of flour [36,37]. All these parameters can contribute to affect the growth rate of organisms, their competitiveness in sourdough fermentation and eventually their impact on product quality. It is thus presumably to suppose that a long fermentation at elevated temperature (i.e., 35 °C) as applied in the present study selected for *L. fermentum* owing to the thermophilic behavior and high acid resistance of this *Limosilactobacillus* species [38]. Consistent with this hypothesis, the cultivable microbiota of sourdoughs fermented at 37 °C was constituted by *L. fermentum* strains exclusively [39]. Notably, the VIP analysis on the metabolomics profile of doughs revealed that in both native and sprouted rye doughs there was an accumulation of mannitol when LAB were added as starters. Conversion of fructose to mannitol by heterofermentative LAB has been reported in sourdough fermentations [39]. Consistent with this, *L. fermentum* UC3641 has in its genome two Open Reading Frames (ORFs) encoding for a NAD(P)H-dependent mannitol/alcohol dehydrogenase [13].

In addition to oligosaccharide semi-quantification, a metabolomics approach was employed to investigate the overall effect of germination and fermentation on the metabolome of rye sourdoughs. The unsupervised HCA analysis of doughs revealed that germination of rye grains played a major role on the metabolic profile than fermentation, which was also supported by the results from the OPLS-DA models. Germination has been previously assessed as a physiological process in which phytohormones can play a critical role on the development and metabolome of rye grains, which may affect further processing, including fermentation [26]. Furthermore, germinated grains show a high biosynthetic potential and promote the activity of hydrolytic enzymes that lead to structural modifications [9], which could reflect in greater accessibility or diversity of fermentative bacteria. We assessed the presence of LAB species that could be differentially associated to either native or sprouted rye flour regardless of the fermentation conditions. Metagenomic data revealed that the species *L. plantarum* was typical of the microbiota of native rye doughs, whereas *L. curvatus* was significantly higher in doughs made with sprouted rye. As for *L. plantarum,* this species is known to metabolize a wide range of different carbohydrates of varying complexity, thanks to its rich repertoire of lytic enzymes [40]. Furthermore, among the significant compounds responsible for the discrimination between native- and sprouted-derived rye doughs obtained from OPLS-DA model, several compounds were spotted as VIP markers, especially primary metabolites as amino acids, peptides, and lipid metabolites.

Concerning phenolics, the accumulation of flavonoids and phenolic acids was mostly modulated by fermentation, with yeast as the sole fermenting agent or in combination with LAB, which agrees with the previous study by Katina et al. [9] who reported increased levels of phenolic compounds after fermentation, especially in germinated rye. In parallel, sourdough fermentation contributed to increase significantly the content of total phenolic compounds, especially phenolic acids and alkylresorcinols, because of the pH reduction caused during fermentation [7]. Such compounds were found in this work as discriminant metabolites associated to LAB fermentation, as it is the case of feruloyl, caffeoyl, and coumaroyl derivatives, thus being in line with the results provided by other authors [10]. Ferulic and *p*-coumaric acids are the most prevalent phenolics attributed to rye, reaching a proportion of about the 95% of total phenolic compounds [41]. Notably, all the LAB starters used in our experimental conditions presented in their genomes ORFs encoding for esterases, phenolic acid decarboxylases and phenolic acid transferases [13], suggesting the possible involvement of these enzymes in the conversion of *p*-coumaric acid and ferulic acid in their esterified derivatives. It is important to note that phenolic acids have been previously reported in their esterified forms with diverse biogenic amines [10] as reflected by our results with spermine, spermidine, and putrescine. Moreover, the same authors reported an increase in the accumulation of flavonoids due to mixed fermentation, agreeing with present findings. Overall, the polyphenols enrichment associated with LAB + SC fermentation may suggest an enhancement of the nutritional value of rye doughs, given the biological activities of these compounds as multifaceted bioactive compounds, acting as antioxidant, anti-inflammatory, antitumor and antimicrobial agents, among other health-promoting properties [42].

In the case of terpenoids, little is known about the effect of fermentation on biosynthesis of these compounds in rye sourdough [43] that were widely identified during the current research as triterpenoids. Nevertheless, the presence of terpenoids may improve the shelf life and safety of these matrices, due to their associated antibacterial properties [44]. Concerning protein-derived metabolites, both germination and fermentation played a critical role on the catabolites determined in rye doughs, which agrees with the existing literature. Germination can increase the total proteolytic activity in rye whereas acidification mediated by both LAB and yeasts in sourdough fermentation triggers cereal protease activity by shifting the dough pH to the optimum of aspartic proteases, which represent the major proteases in rye and wheat [45]. Even more important for proteolysis is the activity of strain-dependent intracellular peptidases of sourdough lactobacilli, which enhances the accumulation of amino acids in fermented doughs providing key sources of nitrogen for yeast growth [40,45]. Thus, all these factors contribute to the plethora of free amino acids and peptides spotted in this work. The amount and type of peptides and amino acids occurring in cereal doughs mostly account for the overall quality of bread in that many of these compounds act as taste-active components and flavor precursors. However, as a result of the proteolytic activity of sourdough starters, non-proteinogenic amino acids, like citrulline or ornithine, were previously spotted [10] as well as in this case. *L. fermentum* is among the *Lactobacillus* species that can convert arginine to ornithine via the arginine-deiminase (ADI) pathway [46]. Notably both native and sprouted rye doughs fermented by sourdough LAB were enriched in γ-glutamyl dipeptides such as γ-glutamylglutamic acid and γ-l-glutamyl-l-pipecolic acid in the present study. Besides being naturally presented in certain foods, the synthesis of γ-glutamyl dipeptides may occur in fermented foods via microbial γ-glutamyl transpeptidases and γ-glutamyl cysteine synthetases. Formation of γ-glutamyl dipeptides in sourdough fermented by *Limosilactobacillus reuteri* was attributed to strain-specific biosynthetic capabilities and consistently improved the sensory attributes of the resulting bread [47]. Lipid metabolites play a minor role on the composition of rye sourdoughs motivated by the low-fat content of rye flour [7]. According to our results, the accumulation of lipid metabolites did not show a clear pattern, regardless of the germination and fermentation conditions, with the exception of lysophospholipids, which were harshly accumulated as a result of mixed fermentation. This finding could be explained by the activity of hydrolytic enzymes, such as lipases and phospholipases acting on di- and tryacylglycerides, which were found to be heterogeneously accumulated during fermentation. These enzymes could be sourced from rye flour as well as sourdough starter microorganisms. Nevertheless, the results reported by Koistinen et al. [10] on the untargeted metabolomic profile of rye sourdoughs indicate that fermentation promoted the accumulation of phosphatidylcholines, whereas oxidized fatty acids were found to be down-accumulated. Remarkably, in the present study, a higher level of hydroxy fatty acids was detected in doughs fermented with sourdough LAB as compared to *S. cerevisiae* alone. It is known that hydratases by sourdough lactobacilli can convert oleic acid, linoleic acid, and linolenic acid to hydroxy fatty acids.

## 5. Conclusions

The results of the present study provide a comprehensive view of multiple compositional changes induced by germination and lactic fermentation in cereal flour, which may have implications for the nutritional value, sensory attributes, and functional characteristics of rye bakery products. Fermentation by selected sourdough lactic acid bacteria in addition to baker’s yeast resulted in lower levels of simple sugars and increased levels of mannitol in the dough system, and could thus represent a relevant strategy to reduce sugar in baked goods. Grain germination promoted the accumulation of maltooligosaccharides, a class of molecules displaying several potential biological capabilities. Overall, the combination of rye germination with the combined fermentation of *S. cerevisiae* and LAB promoted the accumulation of nutritionally important metabolites, such as polyphenols, terpenoids, hydroxy fatty acids, and peptides, which also contribute to the enhancement of the technological and sensorial properties associated with rye flour. Indeed, the integrated information provided by metagenomics and untargeted metabolomics offered new insights into the impact of processing technologies on dough quality, which can guide the design and development of novel, health-promoting rye foods.

## Figures and Tables

**Table 1:** Rye dough parameters measured after 24 h of maturation.

Parameter	Rye flour		Starter			*p*-Value	
		Control	SC	LAB + SC	Starter	Germination	Starter × Germination
pH	Native	4.19 ± 0.11 ^a^	4.57 ± 0.36 ^b^	3.97 ± 0.10 ^a^	0.0005	0.005	0.06
	Sprouted	4.57 ± 0.10 ^a^	5.33 ± 0.01 ^b^	4.03 ± 0.01 ^a^			
TTA (%)	Native	0.39 ± 0.01 ^a^	0.42 ± 0.03 ^a^	0.82 ± 0.06 ^bA^	<0.0001	<0.0001	<0.0001
	Sprouted	0.50 ± 0.02 ^a^	0.56 ± 0.08 ^a^	2.08 ± 0.05 ^bB^			
a_w_	Native	1.02 ± 0.002 ^a^	0.94 ± 0.02 ^b^	0.95 ± 0.01 ^b^	0.0001	0.044	0.026
	Sprouted	0.99 ± 0.01 ^a^	0.94 ± 0.01 ^b^	0.96 ± 0.01 ^ab^			
Dough rise (%)	Native	70 ± 4.24 ^a^	143 ± 4.95 ^b^	220 ± 18.38 ^c^	<0.0001	0.22	0.06
	Sprouted	90 ± 4.24 ^a^	130 ± 4.24 ^ab^	180 ± 28.30 ^b^			
Total LAB (log CFU/g)	Native	9.16 ± 0.04 ^a^	6.70 ± 0.09 ^bA^	10.32 ± 0.38 ^c^	<0.0001	0.008	0.09
	Sprouted	8.94 ± 0.01 ^a^	5.29 ± 0.01 ^bB^	9.53 ± 0.12 ^c^			
Total yeast (log CFU/g)	Native	6.27 ± 0.01 ^a^	8.33 ± 0.10 ^b^	8.24 ± 0.17 ^b^	<0.0001	0.56	0.05
	Sprouted	5.47 ± 0.66 ^a^	8.73 ± 0.03 ^b^	8.34 ± 0.01 ^b^			

Data are mean values ± SD. Statistical analysis was carried out using two-way ANOVA followed by Tukey’s post hoc test. Different superscript letters (a, b, c) in a row indicate significant differences (*p* < 0.05) between starter types within each flour group; different superscript letters (A, B) in a column indicate significant differences (*p* < 0.05) between native and sprouted rye flour within each starter type group.

**Table 2:** Number of OTUs, alpha diversity indexes, and Good’s coverage of sequenced samples.

Samples *	OTUs	Chao1	Shannon	Gini-Simpson	Good’s Coverage
nDLAB + SC1	217	386.5	0.733	0.132	0.991
nDLAB + SC2	348	485.8	1.486	0.296	0.987
nDSC1	194	230.1	2.414	0.610	0.993
nDSC2	236	271.3	3.108	0.696	0.984
nControl1	58	100.0	0.093	0.013	0.998
nControl2	415	449.0	3.995	0.751	0.993
sDLAB + SC1	466	602.5	2.579	0.595	0.981
sDLAB + SC2	454	565.8	2.624	0.600	0.981
sDSC1	327	438.0	1.712	0.278	0.988
sDSC2	417	533.4	2.587	0.444	0.982
sControl1	227	312.1	0.989	0.173	0.993
sControl2	305	430.4	1.199	0.205	0.989
nF1	284	300.8	3.211	0.740	0.998
nF2	504	541.1	4.947	0.884	0.996
sF1	596	737.5	5.014	0.841	0.974
sF2	609	799.0	5.028	0.794	0.967

* nDLAB + SC: native rye–dough–LAB + SC; sDLAB + SC: sprouted rye–dough–LAB + SC; nDSC: native rye–dough–SC; sDSC: sprouted rye–dough–SC; nDControl: native rye–dough–control; sDControl: sprouted rye–dough–control; nF: native rye–flour; sF: sprouted rye–flour.

## Data Availability

Raw sequences of full 16S rRNA gene profiling are accessible through SRA study accession number PRJNA850138, with biosample accession numbers from SAMN29160520 to SAMN29160535.

## Supplementary Materials

The following supporting information can be downloaded at: https://www.mdpi.com/article/10.3390/foods12050998/s1, Figure S1: Automated system for malting rye grains, Table S1: Dataset of annotated compounds by UHPLC/QTOF-MS analysis, Table S2: Dataset of annotated compounds by UHPLC/QTOF-MS analysis, according to their MS2 spectral features, Table S3: List of VIP markers associated with the OPLS-DA model discriminating between germination conditions of rye doughs (sprouted vs. native), Table S4: List of VIP markers associated with the OPLS-DA model discriminating between starters of native rye doughs (SC+LAB vs. SC), Table S5: List of VIP markers associated with the OPLS-DA model discriminating between starters of sprouted rye doughs (SC+LAB vs. SC).
